# Newcastle Disease Virus V Protein Inhibits Cell Apoptosis and Promotes Viral Replication by Targeting CacyBP/SIP

**DOI:** 10.3389/fcimb.2018.00304

**Published:** 2018-09-03

**Authors:** Zhili Chu, Caiying Wang, Qiuxia Tang, Xiaolei Shi, Xiaolong Gao, Jiangang Ma, Kejia Lu, Qingsong Han, Yanqing Jia, Xiangwei Wang, Fathalrhman Eisa Addoma Adam, Haijin Liu, Sa Xiao, Xinglong Wang, Zengqi Yang

**Affiliations:** ^1^Department of Avian Disease, College of Veterinary Medicine, Northwest A&F University, Yangling, China; ^2^Department of Preventive Medicine and Public Health, Faculty of Veterinary Science, University of Nyala, Nyala, Sudan

**Keywords:** Newcastle disease virus, V protein, apoptosis, CacyBP/SIP, viral replication

## Abstract

Newcastle disease virus (NDV) has been classified by the World Organization for Animal Health (OIE) as a notable disease-causing virus, and this virus has the ability to infect a wide range of birds. V protein is a non-structural protein of NDV. V protein has been reported to inhibit cell apoptosis (Park et al., 2003a) and promote viral replication (Huang et al., 2003), however, the mechanisms of action of V protein have not been elucidated. In the present study, a yeast two-hybrid screen was performed, and V protein was found to interact with the CacyBP/SIP protein. The results of co-immunoprecipitation and immuno-colocalization assays confirmed the interaction between V protein and CacyBP/SIP. The results of quantitative-PCR and viral plaque assays showed that overexpression of CacyBP/SIP inhibited viral replication in DF-1 cells. Overexpression of CacyBP/SIP in DF-1 cells induced caspase3-dependent apoptosis. The effect of knocking down CacyBP/SIP by siRNA was the opposite of that observed upon overexpression. Moreover, it is known that NDV induces cell apoptosis via multiple caspase-dependent pathways. Furthermore, V protein inhibited cell apoptosis and downregulated CacyBP/SIP expression in DF-1 cells. Taken together, the findings of the current study indicate that V protein interacts with CacyBP/SIP, thereby regulating cell apoptosis and viral replication.

## Introduction

Newcastle disease (ND), which is caused by the Newcastle disease virus (NDV), is one of the most severe avian diseases and can cause great economic loss to the poultry industry worldwide (Alexander, 2011). ND is a contagious disease, and NDV can infect a wide range of domestic and wild birds and has spread worldwide. NDV is an enveloped virus with a negative-strand RNA genome that encodes at least six viral proteins (Alexander and Senne, 2008). Similar to other paramyxoviruses, NDV encodes additional non-structural proteins, named V protein and W protein. These two non-structural proteins are produced by RNA editing during transcription of the P gene (Steward et al., 1993).

NDV is sensitive to interferon (IFN), and V protein plays an important role in preventing IFN response and contributes to the evasion of host immune response by NDV. Recombinant viruses lacking V protein exhibit increased sensitivity to interferon (IFN) compared to the parental viruses (Huang et al., 2003; Park et al., 2003b). It has been demonstrated that V protein possesses IFN-antagonistic activity, and the C-terminal region of V protein is responsible for this activity (Huang et al., 2003; Park et al., 2003b). In addition, the IFN-antagonistic activity of V protein correlates with the differences in virulence of the viral strains (Alamares et al., 2010). Furthermore, the NDV V protein is associated with viral pathogenesis (Mebatsion et al., 2001; Huang et al., 2003). The infectivity of recombinant NDV mutants lacking V and harboring the NS1 gene of the influenza virus (rNDV V(-)/NS1) is reportedly stronger than that of wild-type NDV (wt rNDV) in human cells, suggesting that V protein plays an important role in restricting the host range (Park et al., 2003a). It is not sure whether V protein could potentially regulate viral replication via IFN-independent mechanisms.

Type-1 IFN can induce cell apoptosis (Pokrovskaja et al., 2005). NDV is known to cause oncolysis by triggering apoptosis; however, recombinant NDV strains are cytotoxic to human tumor cell lines of ecto-, endo-, and mesodermal origin, and the cytotoxicity of these NDV strains against tumor cells is due to multiple caspase-dependent pathways of apoptosis, which are independent of IFN signaling (Elankumaran et al., 2006). In general, apoptosis of host cells can inhibit viral replication, and V protein is known for its anti-apoptotic activity (Park et al., 2003a). Thus, V protein might regulate viral replication by affecting apoptosis of infected host cells, but the exact mechanism for this regulation remains to be determined.

S100A6 protein belongs to the A group of the S100 protein family of Ca2+-binding proteins. As an intracellular protein, S100A6 has been implicated in the regulation of several cellular functions, such as proliferation, apoptosis, the cytoskeleton dynamics, and the cellular response to different stress factors (Donato et al., 2017). CacyBP/SIP was initially discovered in mouse Ehrlich ascites tumor cells as an S100A6 target (Filipek and Wojda, 1996; Filipek and Kuznicki, 1998) and later as a Siah-1-interacting protein (Matsuzawa and Reed, 2001). CacyBP/SIP is involved in protein de-phosphorylation, ubiquitination, cytoskeletal dynamics, and all of these functions have been implicated in a wide range of cellular processes, such as cell proliferation, tumorigenesis, cell differentiation and gene expression (Shi et al., 2014; Topolskawoś et al., 2016). In some tumor cells, CacyBP/SIP has been shown to be involved in cell apoptosis, but the promotion or suppression of cancer apoptosis by CacyBP/SIP may depend on cell type (Chen et al., 2013; Fu et al., 2016; Tang et al., 2016). The role of CacyBP/SIP in DF-1 cell apoptosis and the effects of CacyBP/SIP on NDV replication efficiency in these cells remains to be determined.

The present study evaluated the possible mechanisms of the anti-apoptotic effects of V protein. A yeast two-hybrid (Y2H) system was used to screen for proteins interacting with V proteins from a chicken embryonic fibroblast (CEF) yeast library, and CacyBP/SIP was found to interact with V protein in yeast. The role of CacyBP/SIP in promoting apoptosis and inhibiting viral replication was confirmed in DF-1 cells. Finally, the results indicated that V protein can target and down-regulate the apoptosis promotion function of CacyBP/SIP in DF-1 cells.

## Materials and methods

### Virus

Nine- to eleven-day-old SPF embryonated chicken eggs were obtained from a vendor (JINAN SAIS POULTRY CO., LTD.). F48E9 is a genotype IX velogenic strain found in China and was stored in our laboratory. Viruses were propagated in the allantoic cavities of 9–11-day-old embryonated specific-pathogen-free chicken eggs. The allantoic fluid was harvested from the embryonated chicken eggs and stored at −70°C until further use (Gao et al., 2016).

### Vector construction

The cDNA of NDV was isolated from the allantoic fluid by reverse transcriptase PCR using random primers (GenStar, BJ, China). The high-fidelity enzyme PrimeSTAR Max (TaKaRa, DL, China) was used for the entire amplification. The full-length DNA encoding the V gene was amplified by overlap extension PCR (Jang et al., 2010), and the C- and N-terminal domains (VC and VN) and open reading frames encoding CacyBP/SIP were amplified using normal PCR. The specific primers used for gene cloning are listed in Table 1. Then, then V gene and the C- and N-terminal domains of the V gene were cloned in-frame into the expression vector pCAGEN-Flag (based on the vector pCAGEN; a FLAG tag was inserted in front of the multiple cloning site) by using the restriction sites EcoRI and XhoI; the cloned plasmids were named pCAGEN-Flag-V/VC/VN. For the bait protein, the full-length sequence of the V gene was cloned into the EcoRI and Pstl sites of pGBKT7 to generate pGBKT7-V. The full-length CacyBP/SIP gene was amplified from CEF cells and inserted into pCMV-HA (EcoRI, NotI) to generate pCMV-HA-CacyBP/SIP. The sequences of the primers used are listed in the Table 1.

**Table 1:** Primers and siRNA Sequence information.

**Name**	**Forward primer**	**Reverse primers**
V(clone)	5′-*GAATTC*ATGGCGACCTTTACAGACGC-3′	5′-CTCGAG TTACTTACCCTCTGTGATATCG−3′
VC(clone)	5'-GAATTCATGAAGGGGCCCATGGTTGAGT-3′	Same as V
CacyBP/SIP (clone)	5′- CG*GAATTC*ATGGCTGCTGCGCGTGAGGA -3′	5′-TT*GCGGCCGC*TCAAATATCCATTGGTATGTC′
CacyBP/SIP (Q-PCR)	5′-ACCCATCTCTGTGGAAGGCA-3′	5′-AGGTTCATAAGCCCTTCGCT-3′
Gallus β-actin	5′-TTACCCACACTGTGCCCATC-3′	5′-GGGCACCTGAACCTCTCATT-3′
Caspase3	5′-CCATGGCGATGAAGGACTCT-3′	5′-CCCGCTAGACTTCTGCACTT-3′
Caspase9	5′-GCTTGTCCATCCCAGTCCAA-3′	5′-CAGTCTGTGGTCGCTCTTGT-3′
Bcl2	5′-CTTCCGTGATGGGGTCAACT-3′	5′-AGGTACTCGGTCATCCAGGT-3′
FASL	5′-GAGGTGTTGACCCACGTTGT-3′	5′-AGTTGATGCGCTTGTCCTCC-3′
NDV M gene	5′-AAGAAGCAAATCGCCCC-3′	5′-ACGCTTCCTAGGCAGAG-3′
NC	5′-UUC UCC GAA CGU GUC ACG UTT-3′	5′-ACG UGA CAC GUU CGG AGA ATT-3′
siCacyBP/SIP(183)	5′-GCGGCUUCGUGAUGUUCUATT-3′	5′-UAGAACAUCACGAAGCCGCTT-3′
siCacyBP/SIP (326)	5′- GGGAUCAGUCAGAUAAGUUTT-3′	5′- AACUUAUCUGACUGAUCCCTT-3′
siCacyBP/SIP (644)	5′- GCGAAGGGCUUAUGAACCUTT-3′	5′- AGGUUCAUAAGCCCUUCGCTT-3′
IRF1	5′- TTAGACCTCTCGTCCTGCGA-3′	5′-AGAAGCCTTTCCCCTCAACG-3′
IRF3	5′-TACACTGAGGACTTGCTGGAGGT-3′	5′-AAGATGGTGGTCTCCTGATCC-3′
IFN-α	5′-AACCACCCACGACATCCTTC-3′	5′-AGGCGCTGTAATCGTTGTCT-3′
IFN-β	5′-GCTCACCTCAGCATCAACAA-3′	5′-GGGTGTTGAGACGTTTGGAT-3′
IFN-γ	5′-TGAGCCAGATTGTTTCGATG-3′	5′-CTTGGCCAGGTCCATGATA-3′
NDV-specific reverse-transcription primer	5′-AGGGTTCCCGTTCATTCAG-3′	

### Yeast two-hybrid screening

Y2H screening was performed as previously reported (Gao et al., 2016). The CEF yeast library was generated and stored in our lab. The V gene of NDV F48E9 strain was inserted into pGBKT7 vector to get pGBKT7-V. The pGBKT7-V plasmid (about 100 ng) was transformed into Y2HGold yeast cells as bait (pGBKT7-V) according to Yeastmaker^TM^ Yeast Transformation System 2 User Manual (Clontech, Japan). Before Y2H screening, the bait was tested against the autoactivity and toxicity of V in the absence of a prey CEF library. Briefly, 100 ng pGBKT7-V plasmid, 5 μl yeastmaker carrier DNA (10 μg/μl), 50 μl Y2HGold competent cells in 1.1 × TE/LiAc and 500 μl PEG/LiAc were added into a 1.5 mL tube. After gently mixing, the mixture was incubated at 30°C for 30 min. Then 20 μl DMSO was added into the mixture and the tube was place in a 42^o^C water bath for another 15 min. The cells were collected by centrifuging at 12,000 rpm/min for 15 s and resuspended in YPD plus medium. Followed by a further centrifugation, the cells were resuspend in 1 mL 0.9% NaCl solution. At last, 100 μl of a 1/10 dilution and a 1/100 dilution of the transformants were spread onto SDO plates (SD/–Trp plates), SDO/X plates (SD/–Trp/ X-a-Gal), and SDO/X/A plates (SD/–Trp/X-a-Gal/AbA) to test its autoactivity and toxicity. When the bait was confirmed that it has no autoactive activity and toxic effects, one fresh, large colony (2–3 mm) of the bait strain was picked from SD/-Trp plate and inoculated into SD/-Trp liquid medium to prepare a concentrated Y2HGold (pGBKT7-V) culture (>1 × 10^8^ cells per mL in 4–5 mL SD/-Trp medium). Subsequently, we started mating by combining 1 mL of CEF library aliquot (about 2 × 10^8^ cells per mL) with 4–5 mL concentrated bait strain (pGBKT7-V) in a sterile 2 L flask containing 45 mL of 2 × YPDA liquid medium (with 50 μg/mL kanamycin). The flask was incubated at 30°C for mating by slowly shaking (40 rpm). When zygotes presented post 20 h incubation, the cells were pelleted by centrifuging (1,000 g for min) and resuspended in 10 mL 0.5 × YPDA medium. From the mated culture, We spread 100 μL of 1/10, 1/100, 1/1,000, 1/10,000 dilutions from the mated culture on SD/-Trp plates, SD/-Leu plates, and SD/-Leu/-Trp plates to calculate the number of clones screened and mating efficiency. The remainder of the mated culture was plated on fifty DDO/X/A plates with 200 μL per plate. All the blue colonies that grew on DDO/X/A (double dropout medium lacking tryptophan and leucine and supplemented with X-α-Gal and Aureobasidin A) plates post 3–5 days incubation at 30°C were patched out onto higher stringency QDO/X/A (quadruple dropout medium lacking adenine, histidine, tryptophan, and leucine and supplemented with X-α-Gal and Aureobasidin A) agar plates. The blue colonies were screened three times on QDO/X/A plates to rescue the additional library plasmids and eliminate the false positives. The bait plasmid (pGBKT7-V) and rescued prey plasmids were co-transformed into Y2HGold yeast strain to confirm the interactions in yeast cells. The bait plasmid (pGBKT7-53 or pGBKT7-Lam) was co-transformed into Y2HGold with the prey plasmid (pGADT7-T) to serve as positive and negative controls, respectively. The rescued genuine positive AD/library inserts were further sequenced and aligned using the NCBI BLAST program.

### Cell culture

The DF-1, Vero, 293T, and BHK-21 cells used in this study were stored in our laboratory. All cells were cultured in DMEM (Dulbecco's modified Eagle's medium; Thermo, Waltham, USA) supplemented with 10% fetal bovine serum (FBS; Gibco, Grand Island, USA) (2% FBS for maintaining the culture medium), 100 U/mL penicillin, 0.1 mg/mL streptomycin, 2 mM L-glutamine (Invitrogen, Carlsbad, CA, USA), 1% nonessential amino acids (Invitrogen, Carlsbad, CA, USA), and 0.1 mM β-mercaptoethanol (Sigma, Saint Louis, USA).

### Transfection and viral infection

Plasmid or siRNA was transfected to DF-1 and/or Vero cells using Lipofectamine 2000 (Thermo Scientific, NH, USA) according to the manufacturer's instruction. Briefly, when the cultured cells grow to 50% confluence (48 well plaques or confocal dish used for immunofluorescence; 12 well plaques used for viral infection) or 75% confluence (6-well plaques used for detect apoptosis or western-blot experiment), they were used for transfection. Briefly, DNA (0.4, 1.6, and 4 μg for 48 well, 12 well, and 6 well, respectively) or siRNA (10 pmol, 20 pmol, and 100 pmol for 48 well, 12 well, and 6 well, respectively) were diluted in serum-free Optin MEM medium, and then transfected into cells using Lipofectamine 2000 (for DNA transfection, 1, 4, and 8 μl lipofectamine 2000 was used for 48 well,12 well, and 6 well, respectively. For siRNA transfection, 0.6, 2.4, 4.8 μl lipofectamine 2000 was used for 48 well, 12 well, and 6 well, respectively). The transfection efficiency was calculated at 24–48 h post transfection. When the cultured cells attained a density of 50% (used for viral infection or immunofluorescence) or 75% (used to detect apoptosis or western blot), they were collected for transfection. To overexpress the V, VC and VN proteins, the cells were transfected with pCAGEN-Flag-V/VC/VN or with the empty pCAGEN-FlAG plasmid. To overexpress CacyBP/SIP, cells were transfected with pCMV-HA-CacyBP/SIP or with the empty pCMV-HA plasmid. To reduce CacyBP/SIP levels, the cells were transfected with siRNA obtained from Sangon Biotech (Shanghai, China); this siRNA was developed based on the sequence of chicken CacyBP/SIP. To examine the influence of V, VC, CacyBP/SIP and si-CacyBP/SIP (326) on apoptosis, 24–48 h after transfection of DF-1 and/or Vero cells with a plasmid encoding V, VC, or CacyBP/SIP or with the siRNA si-CacyBP/SIP(326), RNA or protein samples (one well cells were lysed in 250 μl RIPA buffer with the protease inhibitor PMSF on ice for 10 min) were collected for further analysis. To examine the influence of V, VC, CacyBP/SIP, and si-CacyBP/SIP(326) on NDV replication, 24 h after cell transfection, 1 MOI of virus was added to the cell culture medium. At the appropriate time, whole RNA was collected and stored until further analysis.

### Quantitative real-time polymerase chain reaction (Q-PCR)

After overexpressing V, VC, or CacyBP/SIP or knocking down CacyBP/SIP in DF-1 or Vero cells, relative mRNA expression of Bcl2, Caspase3, Caspase9, FasL, NDV-M, IFN-α, IFN-β, IFN-γ, IRF1, IRF3, and CacyBP/SIP was tested by Q-PCR. The cells were lysed using Trizol reagent (TaKaRa, Dalian, China) to obtain total cellular RNA. Subsequently, cDNA was synthesized by reverse transcription using the Prime Script RT reagent kit (GenStar, Beijing, China). Q-PCR was performed by using the RealStar Green Fast Mixture (GenStar, Beijing, China) according to the manufacturer's protocol. β-Actin served as the internal control to normalize the relative expression of each gene. Relative transcript levels were analyzed using the ΔΔCt method. The sequences of the Q-PCR primers are listed in the Table 1.

### Viral plaque

The virus titer was measured by a plaque assay. BHK-21 cells in 24-well dishes were infected with NDV from cell supernatants of different groups (0.1–10 μl per well). After 1 h, the medium was replaced with overlay medium. The overlay medium contained 1% methylcellulose to maintain the culture medium. After 3–5 days, the overlay medium was removed and washed three times in phosphate-buffered saline (PBS). Then, the cells were fixed in 4% PFA for 30 min, washed three times in PBS, and stained with crystal violet.

### Western blot analysis

Groups of proteins were extracted from transfected cells. After boiling for 5 min in 5% SDS-PAGE sample loading buffer, equal amounts of protein (50 μg) samples were separated by 12% SDS-PAGE and transferred to a nitrocellulose membrane. The membrane was blocked for 12 h at 4°C in PBS containing 10% skim milk. Specific antibodies against the FLAG tag (1:2,000, Invitrogen, USA), CacyBP/SIP (1:500, BOSTER, China), Caspase3 (1:300, Bioss, China), Bcl2 (1:500, BOSTER, China), Bax (1:500, BOSTER, China), and GAPDH (1:2,000, Sungene Biotech, China) were dissolved in PBS containing 1% skim milk, and then the membrane was treated with this solution. After incubation with the primary antibodies at 4°C for 12 h, the membrane was washed three times with PBST (PBS contain 0.5% Tween 20). Horseradis peroxidase-conjugated anti-rabbit/mouse IgG (1:3,000, Sungene Biotech, China) was then added. Detection was performed by incubating the membrane with Clarity Western ECL Substrate (Bio-Rad, California, USA), and then exposed the membrane in a dark room by using development and fixing baths. The film was developed artificially, and the results were analyzed using a Tanon-410 automatic gel imaging system.

### Immunofluorescence analysis

Cells were transfected with pCAGEN-Flag-V/VC/VN and/or pCMV-HA-CacyBP/SIP 48 h post transfection, and the staining procedure was performed as previously described (Chu et al., 2015). The specific primary antibodies of rabbit anti-CacyBP/SIP (1:100, BOSTER, China), mouse anti-FLAG (1:500, Invitrogen, USA) or mouse anti-HA (1:500, Invitrogen, USA) were used as appropriate. The secondary antibodies used includes donkey anti-Mouse IgG H&L Alexa Fluor 594 (Invitrogen, Carlsbad, CA, USA) and goat anti-rabbit IgG H&L Alexa Fluor 488 (Abcam, Cambridge, UK).

### Co-immunoprecipitation assays

For co-immunoprecipitation (co-IP) assays, 2 μg of pCAGEN-Flag-V, pCAGEN-Flag-VN, or pCAGEN-Flag-VC and 2 μg of pCMV-HA-CacyBP/SIP were co-transfected into 293T cells (60 mm well, 12 μl Lipofectamine 2000). After 48 h, the cells were washed with PBS and harvested. The assay was performed according to the manufacturer's instruction for the Pierce™ Co-Immunoprecipitation Kit (26149, Thermo, USA). In brief, the AminoLink Plus Coupling Resin was immobilized by 3.5 μg affinity-purified anti-Flag antibody or mouse IgG as control (MA1-91878, thermo, USA) at 37°C for 2 h. Then, 350 μl ice-cold IP Lysis buffer and protein mixture (FLAG-V and HA-CacyBP/SIP) from one 60 mm plate transfected 293T cells were added to immobilization resin and gently mixed for 2 h at room temperature. After washing, the binding samples were eluted in 60 μl elution buffer via centrifuge at 2,000 g for 2 min. Then, added 5X Sample Buffer to sample to make a 1X final solution and boiled the sample at 100°C for 5 min. Cool the samples to room temperature before applying them to the gel. Anti-HA primary antibodies(26183, thermo, USA) were used for immunoblotting.

### Flow cytometry

After transfection with pCAGEN-Flag-V/VC and/or pCMV-HA-CacyBP/SIP or si-CacyBP/SIP mRNA, at 24–48 h post transfection, cell apoptosis was detected by the Annexin V/propidium iodide (PI) staining assay according to the manufacturer's protocol. Briefly, 1 × 10^6^ cells were harvested and washed twice with PBS, and the cells were then suspended in 400 μl of binding buffer followed by incubation with 5 μl of Annexin V for 15 min. Then, 10 μl of PI was added, and the cellular apoptosis rate was analyzed by a FACS Calibur instrument (BD FACSAria™ III, USA).

### Statistical analysis

Statistical analysis was performed with GraphPad Prism5 software (GraphPad Software, Inc., CA, USA). All values are expressed as the means ± SD of three independent experiments. Student's *t*-test and one-way ANOVA were used to evaluate the significance of the differences; *P* < 0.05 was considered statistically significant (Geng et al., 2016).

## Ethics statements

The protocol in this study was approved by the Committee on the Ethics of Animal Care and Use of National Research Center for Veterinary Medicine (Permit 20160313088). All animal works complied with guidelines of the Animal Care and Use Committee of Northwest A&F University after prior approval.

## Results

### Overexpression of V protein in DF-1 and vero cells increased viral replication

**Figure 1:**
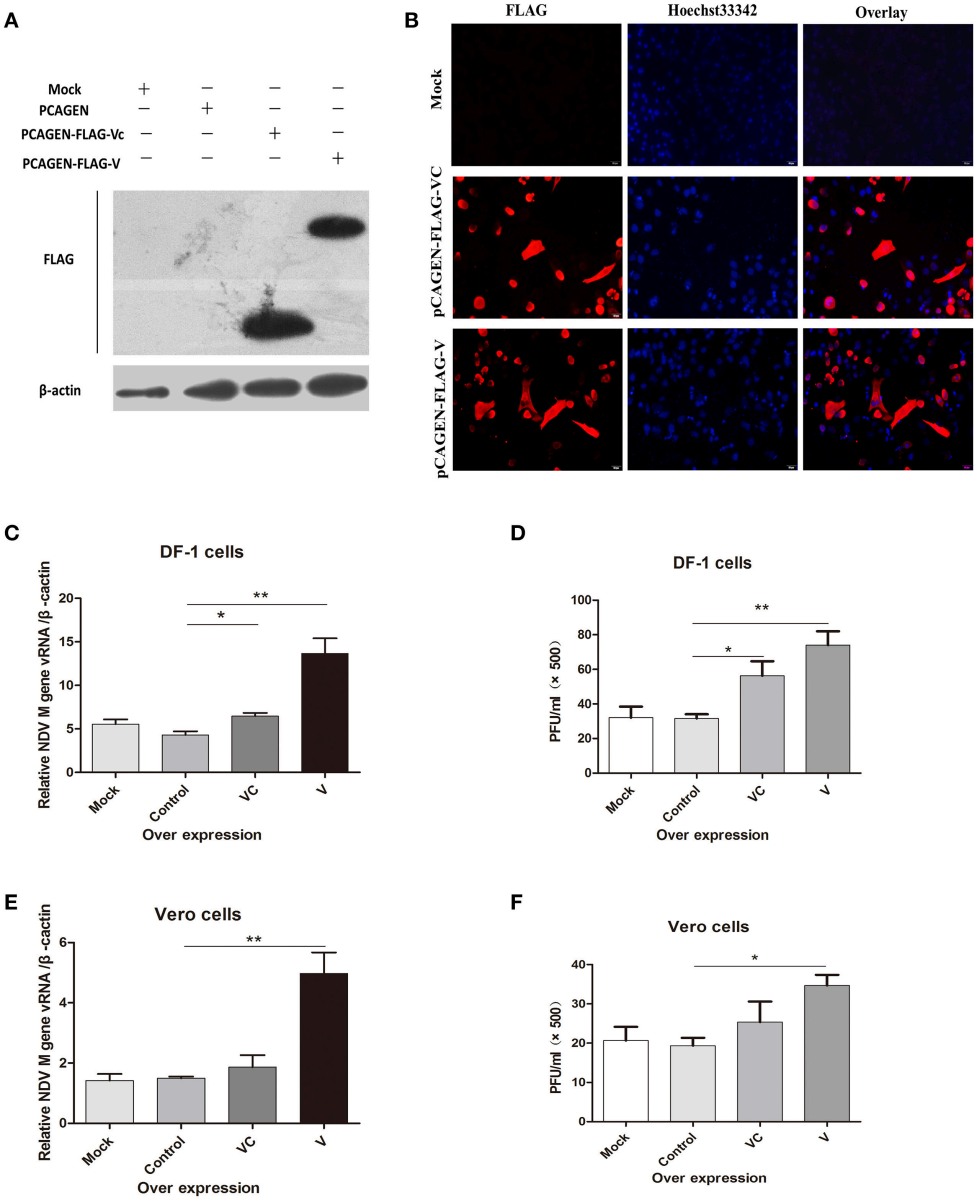
Overexpression of the F48E9 V protein promoted NDV replication in DF-1 and Vero cells. Forty-eight hours after transfection of DF-1 and Vero cells with mock (treated with transfection reagent), control (transfected with pCAGEN), VC (transfected with pCAGEN-Flag-VC), and V (transfected with pCAGEN-Flag-V), **(A)** western blotting and **(B)** immunofluorescence were performed to detect the protein expression of V and VC in DF-1 cells. **(C)** Q-PCR was performed to test the total viral RNA 24 h after V protein overexpression in DF-1 cells. The cells were infected with F48E9 (1 MOI) for an additional 24 h, and **(D)** a viral plaque assay was carried out to test the viral titer in the supernatant of the DF-1 cells(and date from from all four independent experiments, by the geometrical mean of the technical triplicate). **(E)** Q-PCR was performed to test the total viral RNA 24 h after V protein overexpression in Vero cells. The cells were infected with F48E9 (1 MOI) for an additional 24 h, and **(F)** a virus plaque assay was carried out to test the viral titer in the supernatant of the Vero cells. Data are the mean ± SD of triplicate samples from a single experiment and are representative of four independent experiments. *P < 0.05 and **P < 0.01.

Previous studies revealed that the anti-IFN activity of the NDV V protein appears to be located in the carboxy-terminal region of the protein (Park et al., 2003b). To determine whether V protein mediates viral replication only by inhibiting the synthesis of interferon, we overexpressed V protein and VC in DF-1 cells. After 36 h, western blotting (Figure 1A) and immunofluorescence (Figure 1B) showed that both V and VC could be detected in transfected cells. Twenty-four hours after transfection, the DF-1 and Vero cells were infected with NDV (1 MOI). For both DF-1 and Vero cells, the NDV RNA levels in cells overexpressing V protein were significantly higher 24 h post infection than those in the control group (Figures 1C,E). The virus titer in the cell supernatant was measured by a plaque assay, compared with control group, overexpression of V could increase the virus titer in the supernatant by about two-fold, but overexpression of VC could significantly increased the virus titer only in DF-1 cells (Figures 1D,F). Taken together, these results demonstrated that V protein can help viral replication even in cells defective in interferon production, suggesting that V protein may be involved in other mechanisms that promote NDV replication.

### C-terminal domain of NDV V protein targeted the host protein CacyBP/SIP

**Figure 2:**
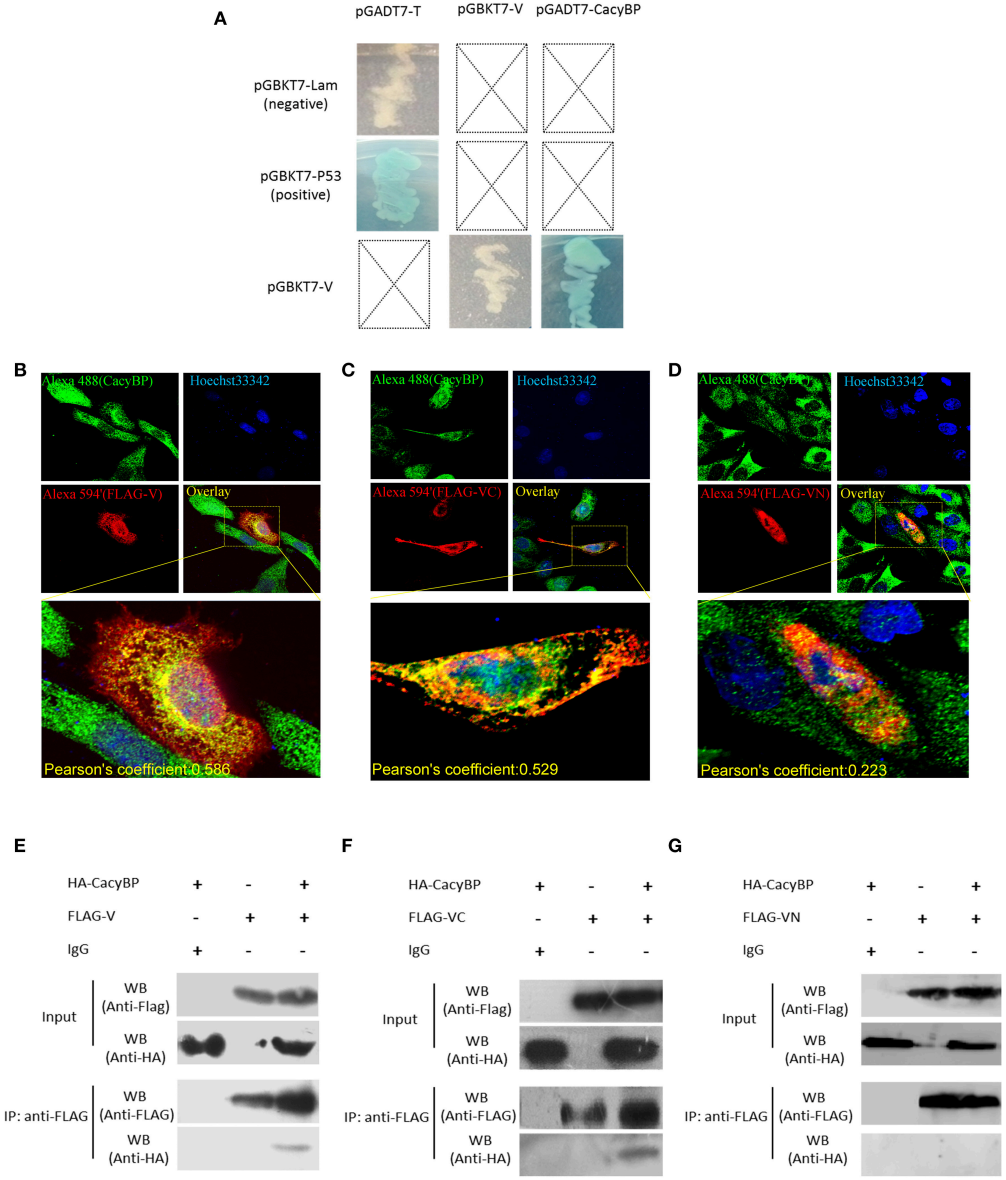
CacyBP bound to C-terminal domains of V protein. **(A)** Identification of protein interaction partners of V protein by yeast two-hybrid screening of a CEF cDNA library. **(B–D)** Co-localization of CacyBP with V, VC, and VN proteins in DF-1 cells. DF-1 cells were plated on coverslips and transfected with Flag-V, Flag-VN and Flag-VC. Forty-eight hours after transfection, the cells were stained with mouse anti-FLAG and rabbit anti-CacyBP antibodies, which was followed by staining with donkey anti-rabbit Alexa Fluor® 488 (green) and goat anti-mouse Alexa Fluor^®;^ 594 (Red) as secondary antibodies. The nucleus was subsequently stained with Hoechst 33342, and the images were captured using an ANDOR Revolution WD confocal microscope. Pearson's coefficient were analysis by Imaris (Microscopy Image Analysis Software, Bitplane, Switzerland), and Pearson's coefficient >0.5 were considered to be probable co-localization. **(E–G)** HEK-293T cells in 60 mm cell culture dishes were co-transfected with the pCAGEN-Flag-V (or Flag-VN or Flag-VC) and pCMV-HA-CacyBP expression plasmids. Transfected cells were harvested and lysed 48 h after transfection, and the experiment was conducted according to the manufacturer's instructions for the Co-Immunoprecipitation Kit. After washing, immunoprecipitated proteins were identified and analyzed by western blotting using anti-HA or anti-FLAG antibodies.

Yeast two-hybrid screening identified 15 proteins (high identity >95% proteins in NCBI) as having potential interactions with NDV V protein (Table 2). The identified proteins were predicted as being involved in RNA binding, cancer-related pathways, and apoptosis-related pathways. Previously, our group focused on the V protein mediated host apoptosis. CacyBP/SIP was reported to participate in apoptosis (Chen et al., 2013; Fu et al., 2016; Tang et al., 2016). Among the 30 clones identified, four clones corresponded to CacyBP/SIP, we chose it for further study due to its involvement in the cell apoptosis adjustment. To identify the target host protein of V protein, V protein was used as a bait protein, and Y2H screening was performed to identify the interaction partner of V protein from the CEF yeast library. First, we verified interactions between CacyBP/SIP and V by the Y2H system. The yeast strain Y2HGold was co-transformed with the prey plasmid PGADT7-CacyBP/SIP and the bait plasmid PGBKT7-V. The bait plasmid (pGBKT7-V) and rescued prey plasmis (pGADT7-CacyBP/SIP) were co-transformed into Y2HGold yeast strain to confirm the interactions in yeast cells. The bait plasmid (pGBKT7-53 or pGBKT7-Lam) was co-transformed into Y2HGold with the prey plasmid (pGADT7-T) to serve as positive and negative controls, respectively. And the co-transformation results showed that CacyBP/SIP rescued from QDO/X/A plates was genuine positive clones (Figure 2A). Meanwhile, both positive (pGBKT7-53 and pGADT7-T co-transformants) and negative (pGBKT7-Lam and pGADT7-T co-transformants) control groups were found eligible. To examine whether the interaction of V protein and CacyBP/SIP occurs in the same cellular compartment, we first performed immunofluorescence experiments by transfecting the pCAGEN-Flag-V plasmid into DF-1 cells. The study showed intense staining for V protein in the cytoplasm and nucleus, and the CacyBP/SIP signals exhibited punctate foci in the cytoplasm and nucleus (partial cells); most overlapping fluorescence spots were observed in cytoplasm. Importantly, co-localization of V protein and VC was observed, the Pearson's coefficient were more than 0.5, and indicating that probable co-localization(V/VC and CacyBP/SIP) (Figures 2B,C), while co-localization of VN was not observed and the Pearson's coefficient were 0.223 (Figure 2D). To map the binding domain of V protein that interacts with CacyBP/SIP, co-immunoprecipitation assays were performed using full-length V protein, VC, and VN. The indicated proteins were produced by expression in 293T cells along with pCMV-HA-CacyBP/SIP. The immunoprecipitated proteins were identified by western blot analysis. The result suggested that V protein can interact with CacyBP/SIP (Figure 2E). VC, but not VN, exhibited similar results as V protein (Figures 2F,G), indicating that the C-terminus of V protein is essential for the interaction with CacyBP/SIP.

**Table 2:** The results of the positive clones mating with V protein BLAST to NCBI.

**Protein no**.	**Protein name**	**Gene**	**NCBI protein accession no**.	**Max identity (%)**	**No. of clones**
1	Calcyclin binding protein (CACYBP)	CacyBP/SIP	XM_422279.6	99	4
2	Rho GDP dissociation inhibitor gamma	ARHGD1G	XM_003642163.4	100	2
3	Citrate synthase	CS	XM_015300289.2	100	2
4	Adenylate kinase 3	AK3	XM_015280190.2	99	1
5	Uncharacterized LOC107049257	LOC107049257	XM_025145642.1	100	7
6	Filamin A interacting protein 1	FILIP1	XM_015284761.2	99	1
7	Elastin microfibril interfacer 1	EMILIN1	XM_015285012.2	100	1
8	WWC family member 3	WWC3	XM_015273752.2	96	3
9	Sarcoglycan beta	SGCB	NM_001031155.1	97	1
10	B-TFIID TATA-box binding protein associated factor 1	BTAF1	XM_015288828.2	98	1
11	Adenylosuccinate synthase	ADSS	XM_015283953.1	95	3
12	Collagen type VI alpha 3 chain	COL6A3	XM_015289155.2	98	1
13	Frizzled-1	cFz-1	AF224314.1	97	1
14	Tet methylcytosine dioxygenase 1	TET1	XM_025151681.1	95	1
15	Heterogeneous nuclear ribonucleoprotein H2	HNRNPH2	XM_015293725.2	99	1

### Overexpression of CacyBP/SIP in DF-1 cells suppressed NDV replication and induced cell apoptosis

**Figure 3:**
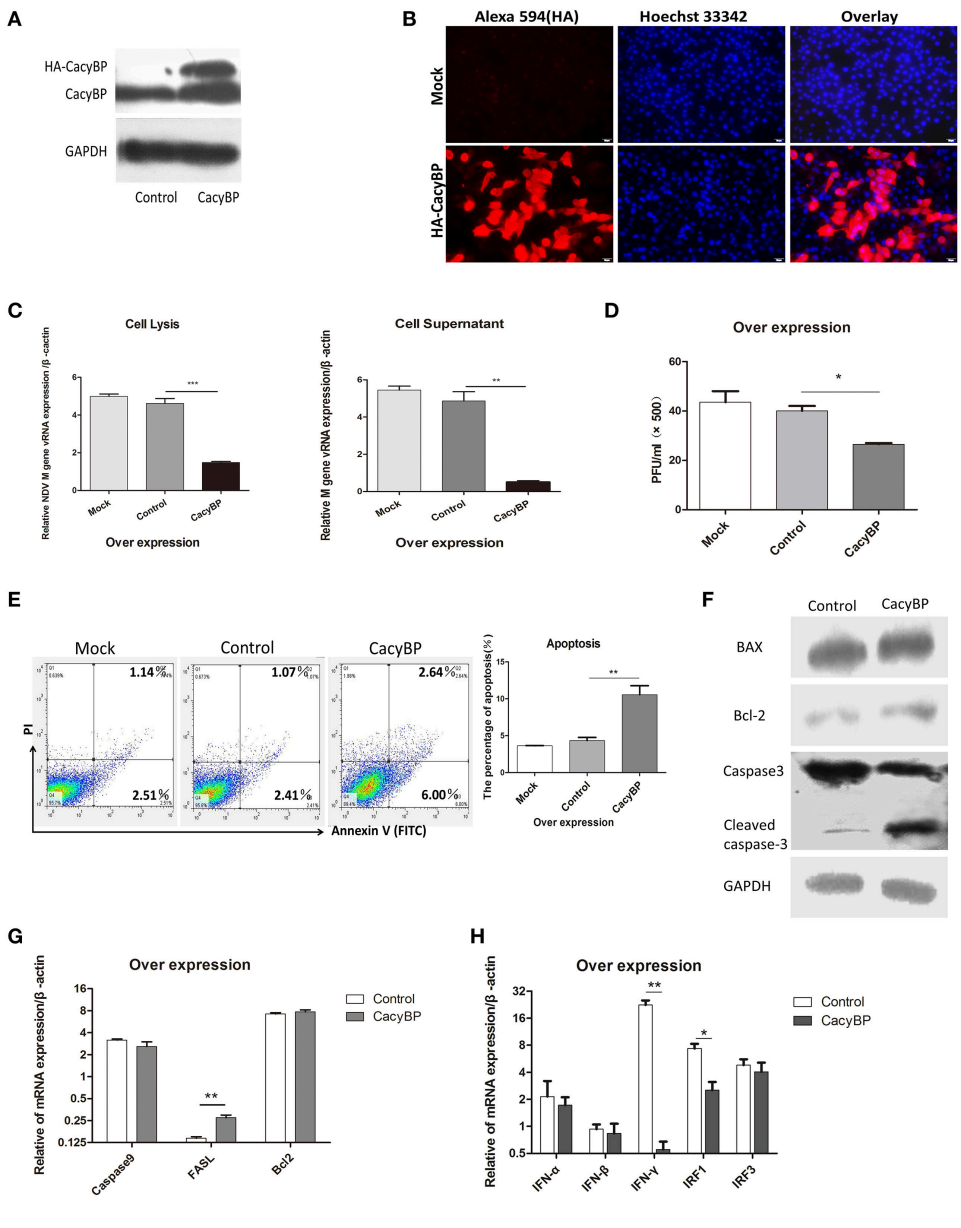
Overexpression of CacyBP in DF-1 cells arrested viral replication and enhanced apoptosis. DF-1 cells were transfected with mock and/or control and CacyBP/SIP and incubated for 48 h. Mock, treated with transfection reagent; control, transfected with pCMV-3HA; CacyBP, transfected with pCMV-3HA-CacyBP/SIP. **(A)** Endogenous and exogenous CacyBP/SIP was detected by western blotting. **(B)** Expression of the 3HA-CacyBP protein in DF-1 cells was detected using immunofluorescence. **(C)** Replication kinetics of NDV RNAs from DF-1 cells. Both cell lysates and supernatants were collected 48 h after transfection (at 24 h after transfection, 1 MOI of F48E9 NDV was inoculated in three groups). Q-PCR was used to measure viral RNA replication. **(D)** Viral plaque formation tests were further used to measure the number of virus particles in supernatants. **(E)** Flow cytometry was used to analyze cell apoptosis. DF-1 cells were transfected with mock and/or control and CacyBP/SIP and incubated for 48 h, an annexin V assay was followed by flow cytometry to monitor for percentage of cells undergoing early apoptosis (bottom right quadrant) and late apoptosis(upper right quadrant).Y-axis is PI signal;X-asis is annexin V-FITC signal, right graph data were the percentage of total apoptosis (bottom and upper right quadrant)and data from three independent experiments. **(F)** The transfected cell lysate was analyzed by western blotting with the indicated apoptosis-related antibody. **(G)** Q-PCR was used to analyze the expression of apoptosis-related markers and **(H)** immune-associated markers 24 h after transfection with pCMV-HA or pCMV-HA-CacyBP. Data shown in **(C,D)** are mean ± SD of four independent experiments in **(E,G,H)** are mean ± SD of three independent expriments. *P < 0.05; **P < 0.01; ***P < 0.001.

To examine whether CacyBP/SIP was associated with NDV replication *in vitro*, pCMV-HA-CacyBP/SIP was transfected into DF-1 cells. Thirty-six hours after transfection, western blotting (Figure 3A) and immunofluorescence (Figure 3B) experiments were performed, and the results suggested that CacyBP/SIP was successfully overexpressed in the DF-1 cells. Then, the CacyBP/SIP-overexpressing DF-1 cells were infected with NDV (F48E9, 1 MOI). The results of the Q-PCR (Figure 3C) and viral plaque (Figure 3D) experiments suggested that overexpression of CacyBP/SIP in DF-1 cells can decrease NDV replication at 24 hpi compared with the control group (empty vector) and the mock group. Previous studies have suggested that overexpression of CacyBP/SIP might inhibit drug-induced apoptosis by enhancing the Bcl-2/Bax ratio in pancreatic cancer cells (Xiong Chen, Apoptosis 2013). The effect of CacyBP/SIP overexpression in DF-1 on cell apoptosis observed by flow cytometry suggested that overexpression of CacyBP/SIP in DF-1 cells promoted apoptosis (Figure 3E).

To further investigate the molecular mechanisms involved in CacyBP/SIP-mediated apoptosis, the expression of Caspase3, Bax, and Bcl-2 was examined in CacyBP/SIP-related transfectants. The expression of cleaved Caspase3, but not of Bcl2/Bax, increased in response to the upregulation of CacyBP (Figure 3F). Q-PCR results suggested that the expression of Caspase3 decreased, and the expression of FASL increased in response to upregulation of CacyBP/SIP (Figure 3G). However, no significant difference was observed in the expression of other genes, such as Caspase9 and Bcl2. IFN-α/β can inhibit NDV replication. To determine whether CacyBP/SIP can induce IFNα/β to reduce NDV replication, immune molecules were tested. The results showed that only IFN-γ and IRF1 levels decreased significantly in CacyBP/SIP-overexpressing cells (Figure 3H). Taken together, these results indicated that CacyBP/SIP induced cell apoptosis via the Caspase3 pathway in DF-1 cells.

### Knockdown of cacyBP/SIP inhibited cell apoptosis and promoted NDV replication

**Figure 4:**
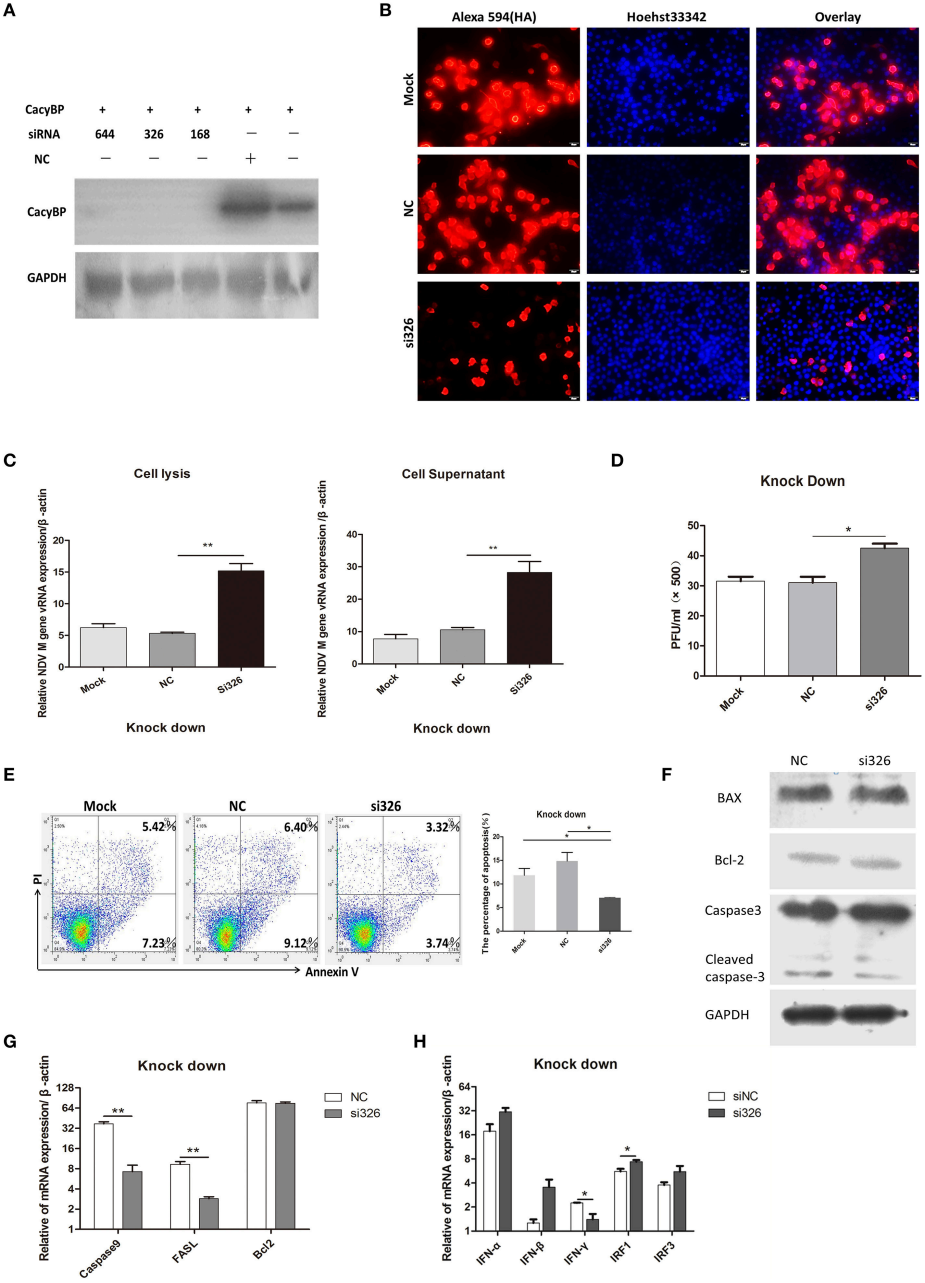
Knockdown of CacyBP facilitated viral replication and arrested apoptosis in DF-1 cells. DF-1 cells were transfected with mock and/or NC and si-CacyBP/SIP and incubated for 36 h. Mock, treated with transfection regent; NC, transfected with si-NC; si183/326/644, separately transfected with those siRNAs targets CacyBP/SIP. **(A)** Endogenous CacyBP/SIP was detected by western blotting. **(B)** DF-1 cells were co-transfected with si-CacyBP (326) and pCMV-HA-CacyBP. Thirty-six hours after transfection, protein expression of HA-CacyBP was detected by anti-HA antibody through immunofluorescence in DF-1 cells. **(C)** Replication kinetics of NDV RNAs from the mock, NC, and siRNA (326) groups of DF-1 cells; 1 MOI of F48E9 NDV were inoculated into the three groups of cells 24 h after transfection. Q-PCR was used to measure viral RNA replication 24 hpi. **(D)** Viral plaque formation tests were further used to measure the number of viruses in the supernatants. **(E)** Three cell cultures were prepared and transfected with mock, NC and siRNA (326), and then, the cells were washed and harvested, an annexin V assay was followed by flow cytometry to monitor for percentage of cells undergoing early apoptosis (bottom right quadrant) and late apoptosis(upper right quadrant). Y-axis is PI signal; X-asis is annexin V-FITC signal. Right graph data were the percentage of total apoptosis (bottom and upper right quadrant)and data from three independent experiments. **(F–H)** Q-PCR was used to analyze the expression of apoptosis-related markers and immune-associated markers 24 h after transfection with NC and si-CacyBP/SIP. **(G)** The transfected cell lysate was analyzed by western blotting with the indicated apoptosis-related antibody. Data shown in **(C,D)** are mean ± SD of four independent experiments, in **(E,G,H)** are mean ± SD of three independent expriments. *P < 0.05 and **P < 0.01.

To test the hypothesis that downregulation of CacyBP contributes to promoting NDV replication, a CacyBP knockdown was generated in DF-1 cells. DF-1 cells were transfected with a pool of four short interfering RNAs (si-CacyBP/SIP), which targeted to CacyBP/SIP mRNA for degradation, and one of them was a negative control (NC). The results of western blotting suggested that all the si-CacyBP/SIP reduced the endogenous expression of the CacyBP/SIP protein (Figure 4A). Then co-transfected DF-1 cells with pCMV-HA-CacyBP/SIP and si-CacyBP/SIP(326), the immunofluorescence results suggested that si-CacyBP/SIP reduced the exogenous expression of the HA-CacyBP/SIP protein (Figure 4B).To examine the effect of knocking down CacyBP/SIP on NDV replication in DF-1 cells, the CacyBP/SIP-knockdown DF-1 cells were infected with NDV (F48E9, 1 MOI). The results of the Q-PCR (Figure 4C) and viral plaque (Figure 4D) experiments suggested that knock down of CacyBP/SIP in DF-1 cells can slightly increased NDV replication at 24 hpi. Overexpression of CacyBP/SIP in DF-1 cells led to cell apoptosis. In addition, cell apoptosis was also tested by flow cytometry 24 h after transfection with si-CacyBP/SIP (326), and the proportion of apoptotic cells in CacyBP/SIP-knockdown DF-1 cells was significantly less than that in the control and mock groups (Figure 4E). To further investigate the molecular mechanisms involved in CacyBP/SIP-mediated apoptosis, the expression of Caspase3, Caspase9, Bcl-2, and FASL was examined in si-CacyBP/SIP-related transfectants. The Q-PCR results suggested that the expressions of Caspase3, Caspase9, and FASL decreased in response to knocking down CacyBP/SIP (Figure 4G).

However, no significant difference was observed in Bcl2 expression. The protein level of cleaved Caspase3 slightly decreased in response to knocking down CacyBP/SIP (Figure 4F). To determine whether knocking down CacyBP/SIP can induce an innate immune reaction and promote replication of NDV, immune-associated molecules were detected by Q-PCR. The results showed that the levels of IFN-γ and IRF1 changed significantly (Figure 4H). Taken together, these results indicated that CacyBP/SIP induced apoptosis via the Caspase3 pathway in DF-1 cells.

### V protein inhibited apoptosis of DF-1 cells and negatively regulated cacyBP/SIP

**Figure 5:**
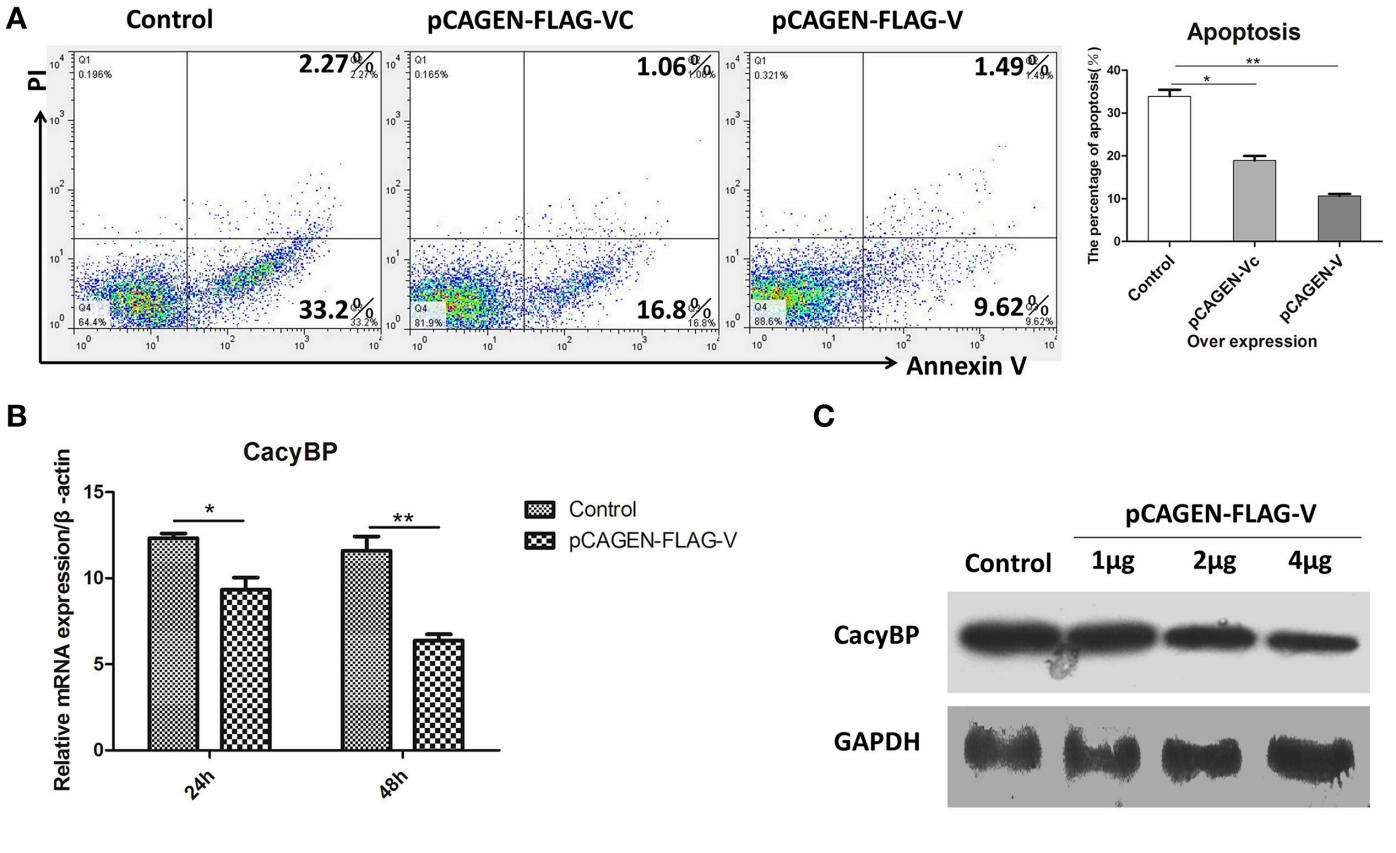
V protein inhibited apoptosis by downregulating CacyBP in DF-1 cells. DF-1 cells were transfected with the control (pCAGEN-Flag), pCAGEN-Flag-VC, and pCAGEN-Flag-V. **(A)** The transfected cells were washed and harvested 48 h after transfection, and flow cytometry was used to analyze cell apoptosis. **(B)** Twenty-four and forty-eight hours after transfection, the mRNA levels of CacyBP were measured by Q-PCR. **(C)** Protein expression of CacyBP was measured in DF-1 cells. The cells were transfected with pCAGEN-Flag (control) and pCAGEN-Flag-V (1, 2, and 4 μg), and after 48 h, the cells were harvested and analyzed by western blotting. Data shown in **(A,B)** are mean ± SD of three independent experiments. *P < 0.05; **P < 0.01.

Homologs of V protein are known to possess anti-apoptotic properties, but the mechanism of inhibition of host cell apoptosis is not entirely understood. NDV V was overexpressed in DF-1 cells, and 48 h later, the cells were collected and stained with Annexin-V-fluorescein (FITC) and PI as per the manufacturer's instructions. The results showed that DF-1 cell apoptosis could be inhibited by V protein (Figure 5A). Furthermore, to determine whether V protein overexpression in DF-1 cells affects the expression of CacyBP mRNA, the mRNA, and protein levels of CacyBP/SIP were evaluated by real-time quantitative PCR and western blotting. Then, 24 and 48 h after transfection, the expression levels of CacyBP/SIP in V-protein-overexpressing DF-1 cells were found to be significantly lower than those in the control group (Figure 5B). Forty-eight hours after transfection (with varying amounts of pCAGEN-Flag-V), the protein levels of CacyBP/SIP decreased to varying degrees along with V protein overexpression (Figure 5C). Taken together, these results suggest that the non-structural protein V can regulate CacyBP/SIP and suppress the apoptosis of DF-1 cells via a Caspase-3-dependent pathway.

## Discussion

The NDV V protein can assist viral replication, and VC is the functional domain (Huang et al., 2003; Park et al., 2003b; Childs et al., 2007; Alamares et al., 2010). In this study, the function of VC and the full-length V protein in NDV replication were evaluated. The results showed that the ability of full-length V protein in promoting NDV replication is stronger than VC in DF-1 cells, suggesting that the integrity of the V protein was important for its function. However, over-expression VC couldn't promote NDV replication in Vero cells. This may due to that the Vero cell was deficency in IFN pathway (Desmyter et al., 1968), so V protein couldn't antagonize IFN by targeting molecules in the interferon pathway. The above result inferred that V protein enhanced NDV replication not only by inhibiting IFN production but also by other mechanisms.

By performing Y2H screening, the study found that V protein targeted the host CacyBP/SIP protein and suppressed the expression of this protein in DF-1 cells. It has been reported that CacyBP/SIP localizes to the cytoplasm and nucleus, and nuclear translocation of CacyBP/SIP can be induced by cell proliferation of gastric cancer, which is promoted by gastrin (Zhai et al., 2014). In this study, we determined that CacyBP/SIP was localized in both the cytoplasm and nucleus. Among all of the NDV structural proteins, the matrix (M) protein is the only one that is reported to be nuclear (Peeples, 1988; Peeples et al., 1992; Duan et al., 2013a,b, 2014). To our knowledge, the subcellular localization of V protein has not been reported. Our results suggested that V protein may be localized in both cytoplasm and nucleus in DF-1 cells.

Apoptosis is an orderly process of cell death, and apoptosis of host cells limits viral replication (Kang et al., 2017). Overexpression of CacyBP/SIP can promote apoptosis in DF-1 cells, while knockdown of CacyBP/SIP by si-CacyBP/SIP can reduce apoptosis in DF-1 cells. Furthermore, overexpression of CacyBP/SIP in DF-1 cells induced apoptosis via a Caspase3-activated pathway and had no significant effect on Bcl2/Bax expression. This study revealed that overexpression of CacyBP/SIP reduced NDV replication and facilitated apoptosis of DF-1 cells, while knockdown of CacyBP/SIP in DF-1 cells downregulated apoptosis and facilitated viral replication.

It has been reported that IFN-γ can induce apoptosis in many kinds of cells, such as leukemia cells (Xia et al., 2017) and epithelial cells (Wu et al., 2016). An unexpected result was observed in the present study; the mRNA of IFN-γ was downregulated by both overexpression and knockdown of CacyBP/SIP in DF-1 cells, indicating that IFN-γ may not play a major role in CacyBP/SIP-induced apoptosis in DF-1 cells. Moreover, CacyBP/SIP had no positive effects on the expression of IFN-α and IFN-β (Figures 3H, 4H), suggesting that the IFN-α/β pathway may not be involves in the effect of CacyBP/SIP on NDV replication. Interferon regulatory factor 1(IRF1) serves as an activator of genes involved in immune response. In this study, overexpression of CacyBP/SIP down regulated IRF1 expression but did not affect the expression of IFN-α/β. Some reports suggests that there was a significantly positive correlation between IRF1 and apoptosis (Kung et al., 2014; Zhang et al., 2016; Zhao et al., 2016), even sometimes not like that (Chapman et al., 2000). This may be IRF1 transcriptional regulator which displays a remarkable functional diversity in the regulation of apoptosis. However, a recent study reported that there is an interplay between CacyBP/SIP and proteins/factors involved in the immune response (Kadziołka et al., 2017). We supposed that some unknown mechanisms may be involved in the effect of CacyBP/SIP and IRF1on the suppression of NDV replication in addition to the induction of cell apoptosis.

NDV can induce both intrinsic and extrinsic caspase-dependent apoptosis pathways (Elankumaran et al., 2006). This study suggested that V protein can reduce the apoptosis of DF-1 cells via downregulation of CacyBP/SIP expression in DF-1 cells. However, CacyBP/SIP can induce caspase3-dependent apoptosis in DF-1 cells and inhibit viral replication, suggesting that CacyBP/SIP is one of the host proteins that regulate cell apoptosis and NDV replication. Determination of whether CacyBP/SIP is the key molecule regulated by V protein to modulate host cell apoptosis requires further investigation.

In summary, this study investigated the role of V protein in regulating host cell apoptosis and viral replication and the potential mechanisms of this regulation. The results showed that V protein can interact with the host cell protein CacyBP/SIP to regulate viral replication by inhibiting host cell apoptosis. Moreover, further studies are required to determine whether other host proteins interact with V protein and participate in the regulation of NDV replication. To clearly understand the function of V protein, a map of the interactions among host proteins and V protein should be constructed in future studies.

## Author contributions

ZY, ZC, CW, and XG designed research. ZC, CW, XG, QT, JM, FA, HL, and KL performed research. ZC, QH, YJ, XiaW, FA, CW, XinW, SX, ZY, and XS analyzed data. ZC and ZY wrote the paper.

### Conflict of interest statement

The authors declare that the research was conducted in the absence of any commercial or financial relationships that could be construed as a potential conflict of interest.
